# Supplementary material for: Complexome profiling of the Chlamydomonas psb28 mutant reveals TEF5 as an early PSII assembly factor
Source: Plant Cell. 2025 Mar 17;37(6):koaf055. doi: 10.1093/plcell/koaf055 (PMC12164590; doi:10.1093/plcell/koaf055)
Supplement: koaf055_Supplementary_Data [file koaf055_supplementary_data.zip › psb28_interactive.zip!/plots/intensity/qualityPlots/peptides/METH_Cre06.g250902.t1.1.html]

### Cre06.g250902.t1.1

Trivial name: METH  
SeqCoverage [median]: 18.80  
Mass: 267.83 kDa  
  
 

| Sequence | MissedCleavage | Mass [Da] | wtIntensity | muIntensity | Score |
| --- | --- | --- | --- | --- | --- |
| AAMYALEK | 0 | 895.45 | 476.60 | 0.00 | 81.78 |
| DGVKPTAAFVPLDK | 1 | 1456.79 | 0.00 | 589.98 | 47.77 |
| FTLEEEDFRGER | 1 | 1526.70 | 1264.91 | 4309.29 | 48.00 |
| FVAGAIGPTNK | 0 | 1073.59 | 6143.18 | 9799.69 | 96.67 |
| FVNLLVSDPEISR | 0 | 1487.80 | 2631.16 | 0.00 | 122.26 |
| IIFIDGAMGTQIQK | 0 | 1533.82 | 5106.73 | 5164.40 | 130.10 |
| KPVTVPPLMR | 1 | 1136.67 | 0.00 | 743.75 | 42.80 |
| LNPIPAGKDQLAPDAKPAPVPK | 2 | 2236.26 | 643.82 | 3657.08 | 48.66 |
| LPVFVSGTIVDNSGR | 0 | 1559.83 | 351.99 | 3264.37 | 91.94 |
| LSGLEPLNYTPDASNMRR | 1 | 2033.00 | 676.88 | 1578.61 | 56.51 |
| LSVDDMLKVK | 1 | 1146.63 | 6471.39 | 3506.07 | 50.35 |
| TAAQLAK | 0 | 701.41 | 733.93 | 0.00 | 100.07 |
| TLSVSPSVENPAFR | 0 | 1502.77 | 7908.66 | 11358.95 | 152.07 |
| VSGGVSNIAFSFR | 0 | 1339.69 | 522.87 | 0.00 | 93.26 |
|  |  |  | 32932.1 | 43972.2 |  |
